# Deletion of JMJD2B in neurons leads to defective spine maturation, hyperactive behavior and memory deficits in mouse

**DOI:** 10.1038/tp.2016.31

**Published:** 2016-03-29

**Authors:** K Fujiwara, Y Fujita, A Kasai, Y Onaka, H Hashimoto, H Okada, T Yamashita

**Affiliations:** 1 Department of Molecular Neuroscience, Graduate School of Medicine, Osaka University, Suita, Japan; 2 Japan Science and Technology Agency, CREST, Tokyo, Japan; 3 Laboratory of Molecular Neuropharmacology, Graduate School of Pharmaceutical Sciences, Osaka University, Suita, Japan; 4 iPS Cell-based Research Project on Brain Neuropharmacology and Toxicology, Graduate School of Pharmaceutical Sciences, Osaka University, Suita, Japan; 5 Molecular Research Center for Children's Mental Development, United Graduate School of Child Development, Osaka University, Suita, Japan; 6 Department of Biochemistry, Kinki University Faculty of Medicine, Sayama, Japan

## Abstract

JMJD2B is a histone demethylase enzyme that regulates gene expression through demethylation of H3K9me3. Although mutations of JMJD2B have been suggested to be responsible for neurodevelopmental disorders, the function of JMJD2B in the central nervous system (CNS) remains to be elucidated. Here we show that JMJD2B has a critical role in the development of the CNS. We observed JMJD2B expression, which was especially strong in the hippocampus, throughout the CNS from embryonic periods through adulthood. We generated neuron-specific JMJD2B-deficient mice using the cre-loxP system. We found an increase in total spine number, but a decrease in mature spines, in the CA1 region of the hippocampus. JMJD2B-deficient mice exhibited hyperactive behavior, sustained hyperactivity in a novel environment, deficits in working memory and spontaneous epileptic-like seizures. Together these observations indicate that JMJD2B mutant mice display symptoms reminiscent of neurodevelopmental disorders. Our findings provide evidence for the involvement of histone demethylation in the formation of functional neural networks during development.

## Introduction

Neurodevelopmental disorders are an essential and poorly understood clinical challenge. The complications they cause such as learning disorders, memory-formation deficits and epilepsy, have profound effects on patients' daily life.^1, 2^ Numerous gene mutations are involved in these pathologies, including genes associated with postsynaptic components.^3, 4^ Moreover, recent studies provide increasing evidence of the importance of epigenetic mechanisms in the underlying pathobiology,^5^ and mutations of genes involved in epigenetic regulation are found in patients with neurodevelopmental disorders.^6, 7^ In addition, recent report suggests that several histone-modification genes, including both histone methyltransferases and demethylases, are causative risk genes for such disorders.^8^ Nevertheless, our knowledge of the involvement of histone modifications in neurodevelopmental disorders is limited.

Histone modification is an epigenetic process that regulates gene expression through the modulation of chromatin structure. An important histone-modification process is histone methylation and demethylation. Among the best understood and most essential classes of histone demethylases is the *Jumonji* family, first cloned by Takeuchi *et al.*^9^ in 1995, which was followed by the identification of the *Jmjd2* family, a *Jumonji* family subgroup. The JMJD2 family consists of 6 subtypes (JMJD2A-JMJD2F); JMJD2B acts as a transcriptional activator of targeted genes through demethylation of specific lysine residues on H3K9me3.^10, 11^ JMJD2B is located in the nucleus in dividing cells and has demethylase activity to H3K9me3.^12^ Previous research has mainly focused on the oncogenic potential of JMJD2B. In particular, overexpression of JMJD2B exhibits oncogenic activity in various human cancers, including prostate, breast, colon and gastric cancers.^13, 14, 15, 16, 17^ In addition, JMJD2B was found to be necessary for self-renewal of embryonic stem cells and induced pluripotent stem cell generation.^18^

The *Jumonji* family appears to also play important roles in the brain. Two mutations of *JMJD2B* have been found in individuals with autism spectrum disorder (ASD) along with other histone demethylase enzymes, including *JMJD1A* and, *JARID1B*.^8^ In addition to these enzymes, mutations of other histone demethylase genes including KDM6A, which is implicated in Kabuki syndrome,^19^ and JARID1C, which is implicated in X-linked mental retardation syndrome,^20^ are found in individuals with neurodevelopmental disorders. Therefore, histone demethylation appears to be critically associated with neurodevelopmental disorders; however, the function of the *Jumonji* family in the central nervous system (CNS) remains largely unknown.

In the present study, we investigated the function of JMJD2B in the CNS by generating neuron-specific, JMJD2B-deficient mice. These mice exhibited various symptoms reminiscent of neurodevelopmental disorders, and impaired synaptic maturation in the hippocampus. These observations indicate that JMJD2B is required for neuronal circuit formation, and its deficiency leads to behavioral and physiologic abnormalities observed in neurodevelopmental disorders.

## Materials and methods

### Mice

For all experiments, the mice were of the C57B6/J strain. *Jmjd2b* floxed mice, in which exon 5 of the *Jmjd2b* allele was flanked by loxP sequences bilaterally,^13^ were crossed with mice expressing Cre recombinase under the control of the *Tau* promoter,^21^ resulting in the specific deletion of JMJD2B in neurons. Both genders were used in all experiments except for behavioral analysis (male mice). In all cases, pups were weaned on postnatal day (P) 28, and housed in sex-matched groups in standard mouse cages under a 12-h light/dark cycle according to the protocols approved by Institutional animal care and Use Committee of Osaka University.

### *In situ* hybridization

Mice were harvested for *in situ* hybridization analysis at embryonic days (E) 13, 15 and 17, and P1, 3, 5, 7, 14 and 56 (8 weeks; *n*=1 per age). All mice were decapitated under deep anesthesia and brains were quickly put into dry ice powder as fresh frozen samples. P0 mice were used for expression analysis and the whole body was quickly put into dry ice powder as fresh frozen samples. The whole brain and whole body were cut into 14-μm-thick sagittal sections using a cryostat and mounted on APS-coated glass slides (Matsunami, Osaka, Japan). Probes for JMJD2B were generated from complementary DNA (cDNA) fragments of mouse DNA by reverse transcription–PCR. We prepared two probes to confirm the specificity. The primer sequences for each probe were as follows: Probe1—forward, 5′-ACGGCAGACGTATGATGACA-3′; reverse, 5′-AAGGGGATGCCGTACTTCTT-3′: Probe2—forward, 5′-TGGTGACTCACAGGGAACAA-3′; reverse, 5′-AACATGTCCAGCCCTCTTTG-3′.

Using these primers, we amplified the target probe sequences by PCR and subsequently integrated the PCR products into the pGEM-T easy vector (Promega, Tokyo, Japan). DNA templates were linearized by restriction enzymes (*Nde*I and *Nco*I) by incubation at 37 °C for 16 h. Complete digestion of the vector was confirmed by agarose electrophoresis.

Then, we prepared solution containing 1 μg template DNA, 1 × concentration DIG RNA labeling mix 2 μl, 5 × transcription buffer, 100 mm DTT and the appropriate RNA polymerase (T7 or SP6). RNA was synthesized by incubation at 37 °C for 2 h. Then, DNase was added and samples were incubated at 37 °C for 10 min followed by RNA probe extraction by ethanol precipitation.

Before beginning hybridization, the sections were air-dried for 1 h and immediately put into 4% paraformaldehyde for 30 min, followed by washing in 0.1% activated DEPC water (not autoclaved) twice for 15 min. Then samples were equilibrated in 5 × SCC for 15 min. Next, the sections were pre-hybridized at 58 °C for 1 h in hybridization buffer (5 × SCC 400 μg ml^−1^, heat-denatured salmon sperm DNA and 50% formamide) and subsequently hybridized at 58  °C for another 40 h in the same solution, but with 400 ng ml^−1^ RNA probe added. Then, sections were rinsed in 2 × SSC at room temperature for 30 min, followed by a wash in 2 × SSC for 1 h at 65 °C, and then in 0.1 × SSC at 65 °C for another hour. Next, sections were washed in buffer A (100 mm Tris-HCl pH 7.5, 150 mm NaCl) at room temperature for 5 min.

Sections were reacted with AP-conjugated anti-Dig Fab antibody (Roche, Mannheim, Germany; dilution 1:5000 in wash buffer A) at room temperature for 2 h. After washing the sections with buffer A twice for 15 min each, the sections were washed with buffer B (100 mm Tris-HCl pH 9.5, 150 mm NaCl, 100 mm MgCl_2_) for 5 min. Next, these sections were incubated with color reagent (NBT 45 μl, BCIP 35 μl, each in 10 ml buffer B) at room temperature for 48 h. After that, sections were washed with buffer B and the reactions were stopped by applying TE buffer (10 mm Tris-HCl pH 8.0, 1 mm EDTA). Finally, sections were dehydrated in an ethanol gradient (70, 80, 95 and 100%) and mounted with EUKITT (O. Kindler, Freiburg, Germany).

### Immunohistochemistry followed by *in situ* hybridization

Immunostaining with DAB as the substrate for horseradish peroxidase followed by *in situ* hybridization was performed according to a previously described method (*n*=1).^22^ After *in situ* hybridization by using fresh frozen sections was complete, sections were washed with buffer B as described above. Then, sections were washed with PBS three times for 5 min each. Next, sections were incubated with NeuN (anti-mouse NeuN 1:100 MAB377, Millipore, Darmstadt, Germany) or GFAP (anti-mouse GFAP 1:500, sc58766, Santa Cruz Biotechnology, Dallas, TX, USA) antibodies diluted in PBS overnight at 4 °C followed by washing with PBS three times at room temperature. Then, sections were incubated with biotin-conjugated anti-mouse-IgG antibody for 30 min in a moisture chamber followed by washing with PBS twice at room temperature. Next, sections were reacted to Avidin–biotin complex using an ABC kit (Vector Laboratories, San Francisco, CA, USA) for 30 min at room temperature in a moist box followed by washing with PBS twice at room temperature. Then, sections were transferred to 50 mm TBS (pH 7.4) for 5 min and reacted in 50 mm Tris buffer (pH 7.6) containing 0.02% diaminobenzidine tetrahydrochloride (Sigma, Tokyo, Japan) and 0.01% hydrogen peroxide for several minutes until the signal was detected. Reactions were stopped by transferring the sections to 50 mm TBS at room temperature for 5 min. Finally sections were dehydrated in an ethanol gradient as described for the *in situ* hybridization method (70, 85, 95 and 100%) and mounted with EUKITT.

### Genotyping

Genomic DNA was prepared from a tail biopsy of each mouse. Tail biopsies were incubated at 55 °C for several hours with 500 μl genotyping lysis buffer containing 0.01 m Tris-HCl (pH 8.0), 0.05 m EDTA (pH 8.0), 0.1 m NaCl, 0.5% SDS and 0.1 mg ml^−1^ proteinase K (Roche, Tokyo, Japan) for several hours and crushed by vortex with 410 μl of 3.75 m NaCl. Then samples were put into box with crushed ice for 10 minutes followed by centrifugation for 10 min at 15 000 r.p.m. Then, 500 μl of supernatant were collected and mixed with 1000 μl of 99.5% ethanol (Wako, Osaka, Japan) followed by centrifugation at 15 000 r.p.m. for 15 min. The supernatant was discarded, 70% ethanol was added to the DNA pellet, and samples were centrifuged for 5 min at 15 000  r.p.m. This supernatant was discarded and the pellet was dehydrated by evaporation at room temperature. Finally, the dry pellet was dissolved in RNase free water (Ambion, Foster, CA, USA).

PCR was performed using KOD FX Neo (TOYOBO, Osaka, Japan) according to the manufacturer's protocol. PCR protocol was as follows; *jmjd2b*; 98 °C 2 min: 1 cycle; 98 °C, 10 s, 57 °C, 15 s, 68 °C, 1 min: repeat for 30 cycles; 72 °C 10 min: 1 cycle. *Tau-cre*; 96 °C, 5 min: 1 cycle; 96 °C, 45 s, 60  °C, 30 s, 72 °C 30 s: repeat for 30 cycles; 72 °C, 30 s: 1 cycle. The primer sequences were as follows: *jmjd2b* forward: 5′-AAGAAGGGTGGGGTGGCGAA-3′; jmjd2b reverse: 5′-CGCCACGTCCTATGGACAATG-3′; Cre forward: 5′-AGGTTCGTTCTCTCATGGA-3′; Cre reverse: 5′-TCGACCAGTTTAGTTACCC-3′; mTau forward: 5′-TATGGCTGACCCTCGCCAGGAGTTT-3′; mTau reverse: 5′-GTCCACCCCACTGACCTTTTAAGCC-3′.

### Real-time PCR

The hippocampi of P0 mice and adult mice (12 weeks) from each genotype (wild type (WT): *n*=3 knock out (KO): *n*=3) were dissected and crushed with an electric mixer in 1 ml TRIZOL reagent (Ambion) followed by centrifugation at 15 000 r.p.m. for 10 min. Then, 100 μl of chloroform were added to the 500 μl of supernatant. The mixture was vortexed for 15 s and left at room temperature for 15 min. Samples were centrifuged at 4 °C for 15 min and subsequently 100 μl of upper water phase were extracted and mixed with 80 μl isopropanol. Samples were left at room temperature for 10 min and centrifuged at 4 °C for 10 min. Supernatant was removed and 70% ethanol was added, followed by centrifugation at 4 °C for 3 min. Finally, the supernatant was removed and the RNA pellet was dried by evaporation and dissolved in 50 μl of RNase free water. We then performed reverse transcription (High Capacity cDNA Reverse Transcription Kit; Applied Biosystems, Foster, CA, USA) on the RNA samples to obtain cDNA fragments. Gene expression levels were measured by real-time PCR (Quant Studio 7Flex, Applied Biosystems) using Taqman probes.

### Golgi staining

Eight-week-old mice of both genotypes were used for Golgi analysis. All mice were decapitated under deep anesthesia and brains were quickly put into dry ice powder as fresh frozen samples. Tissue was stained with the FD Rapid Golgistain Kit (Cat#: PK401, FD Neurotechnologies, Colombia, MD, USA) according to the manufacturer's protocol. Briefly, tissues were immersed in impregnant solution (solution A+solution B) for 14 days in dark box at room temperature with solution exchange after 1 h and 24 h, respectively. Then tissues were transferred to solution C for 5 days with solution exchange after the first 24 h. Tissues were immediately moved into dry ice powder and cut into 200-μm-thick coronal sections using a cryostat. The cut sections were floated in PBS and placed on double gelatin-coated glass slides. Sections were allowed to dry by evaporation at room temperature for 24 h and then moved into a mixture solution containing one part solution D, one part solution E and two parts of MilliQ-water for 7–10 min. Then sections were washed in MilliQ-water (Millipore) (four washes of 5 min each), dehydrated by immersing the section in Histoclear (National Diagnostics, Atlanta, GA, USA) three times (10 min each time) and coverslipped. Analysis was performed using a Neurolucida system (MBF Bioscience, Chiba, Japan). Dendrites of pyramidal neurons located 120–300 μm from the soma were selected. We counted spine numbers and classified spine class along the first 20 μm of each dendrite. We defined mature spines as mushroom-like spines in which the head diameter was wider than the neck diameter. We used five mutant mice and six WT littermates and analyzed four to seven dendrites per animal, mainly in the dorsal CA1 region. We selected secondary dendrites located 120–300 μm from the somas of the pyramidal neurons in CA1, which corresponds to the area of CA1 that receives inputs from Schaffer collaterals from CA3.^23^ We used 3 mutant mice and 3 WT littermates and analyzed 6–10 apical dendrites of pyramidal neurons per animal in layer 2/3 of somatosensory cortex.

### Hippocampal neuron culture and analysis of neuron morphology

The hippocampus was isolated from P1 mice (*n*=4: each genotype). Neurons were cultured in DMEM/F12 medium supplemented with 10% FBS for 1day, and subsequently DMEM/F12 supplemented with B27 (Invitrogen, Foster, CA, USA) for another 6 days in a 37 °C incubator containing 5.0% CO_2_. The neuron density was 4.0 × 10 cells per 3.5-cm dish. After 7 days of incubation, samples were fixed by 4% paraformaldehyde for 30 min followed by incubation in 5% BSA containing 0.3% Triton X-100 for 1 h at room temperature.

Samples were stained with anti MAP2 antibody (anti-rabbit MAP2 1:1000, 45425, Cell Signaling, Danvers, MA, USA) for 16 h at 4 °C. And then samples were washed in PBS with Tween 20 three times followed by incubation with a fluorescence-conjugated secondary antibody (anti-rabbit IgG, 1:1000, Alexa, Invitrogen) for 1 h at room temperature in a dark box. Neuron morphology was traced by Neurolucida. We selected labeled neurons with little or no adjacent stained glia for analysis.

### Timm staining

Mice (3–5 months old) of both genotypes were deeply anesthetized and transcardially perfused with PBS followed by 4% paraformaldehyde in PBS (WT: *n*=3 KO: *n*=3). Following perfusion, fixed samples were post-fixed in the same fixative solution at 4 °C for 16 h and immersed in 30% sucrose in PBS at 4 °C until the tissue sank. Tissues were cut into 40-μm-thick coronal sections using a cryostat and placed on MAS-coated glass slides. Cut sections were dried by evaporation and immersed in 1% sodium sulfide solution (Wako) for 10 min. Then, the sections were fixed again in 4% paraformaldehyde followed by washing in PBS twice. The sections were dried by evaporation and reacted with Timm staining solution containing 30% gum Arabic solution (Wako), 1.7% hydroquinone solution (Wako), 0.08% caustic silver solution (Wako) and 0.2 m citrate buffer solution (pH 4.2; Wako) at room temperature for 2 h in a dark box. The reaction was terminated by immersion in tap water for 5 min. The sections were counterstained with cresyl violet solution for 7 min at room temperature. Finally, sections were dehydrated by ethanol (70, 80, 95, and 100%) and mounted with EUKITT.

### Nissl staining

P0 mouse brains of both genotypes (WT: *n*=3; KO: *n*=3) were fixed in paraformaldehyde at 4 °C for 16 h and impregnated with 30% sucrose until the tissue sank. Then, fixed samples were immediately moved into dry ice powder and cut into 30-μm-thick saggital sections using a cryostat and placed on MAS-coated glass slides. Cut sections were impregnated with Cresyl violet acetate (C5042-10G, Sigma) at room temperature for 10 min and dehydrated in 100% ethanol for 5 min.

### Western blotting

Hippocampal brain lysates of both genotypes of 3 or 4-month-old mice (WT: *n*=3 KO: *n*=3) were boiled in sample buffer for 5 min. The proteins were separated by SDS–PAGE and transferred onto polyvinylidene difluoride membranes (Millipore). The membrane was blocked with 5% not-fat dry milk in PBS containing 0.05% tween-20 (PBS-T) and incubated for 1 h at room temperature or overnight at 4 °C, with PSD95 antibody (anti-mouse PSD95 1:500, MA1-046, Thermo Scientific, Waltham, MA, USA) or GAPDH antibody (anti-rabbit GAPDH 1:1000, sc-25778, Santa Cruz Biotechnology) diluted in PBS-T containing 1% non-fat dry milk. After washing in PBS-T, the membrane was incubated with a horseradish peroxidase-conjugated anti-mouse IgG or anti-rabbit IgG antibody (1:5000, Cell Signaling Technology). For detection, an ECL chemiluminescence system (GE Healthcare) was used. Signals were detected and quantified using the LAS-3000 Amersham Imager 600 (GE Healthcare).

### Behavioral analysis

Adult (8 weeks) WT and JMJD2B mutant male mice were subjected to a battery of behavioral tests. For each test, *n*=8–13 mice per genotype were tested. In each behavior test, we analyzed the mice that were able to complete each behavioral task.

### SHIRPA 1st screening

Initial behavioral and physical screening was performed using SHIRPA (SmithKline Beecham, Harwell, Imperial College Royal London Hospital, Phenotype Assessment), as previously described.^24^

### Locomotor activity

Locomotor activity in an open field was evaluated according to a previously reported method.^25^ Briefly, each mouse was placed in the center of an open, transparent, acrylic cubic box with a black, plexiglass floor (45 × 45 × 30 cm), and allowed to freely explore the environment for 60 min under dim light (3 lux). The distance moved in the first 10 min was defined as 100% in each genotype, and the ratio of the distance moved (%) was assessed. The ambulation of mice was monitored using the Panlab Infrared Actimeter System (LE8815) with acquisition software Acti-Track 2.65 for Windows (Panlab, Barcelona, Spain). Locomotion in the home cage was monitored for 5 days, using a digital counter system with an infrared sensor (Supermex; Muromachi Kikai, Kyoto, Japan) as described previously.^25^

### Y-maze test

The Y-maze test with spontaneous alternation performance was performed as described previously.^26^ Each arm (33 × 13 × 30 cm) converged in an equilateral triangular central area. Each mouse, naive to the maze, was placed at the end of one arm and allowed to move freely through the maze during an 8-min session. The series of arm entries was recorded. Events were counted as entry when the hind paws of the mouse had completely entered the arm. Alternation was defined as successive entries into the three different arms on overlapping triplet sets. The percentage of spontaneous alternations was calculated as the ratio of actual to possible alternations (defined as the total number of arm entries−2) multiplied by 100, as shown in the following equation: alternation (%)=number of alternations/(total arm entries−2) × 100.

### Elevated plus-maze test

The elevated plus-maze test was carried out according to a previously reported method.^27^ The apparatus consisted of four arms (30 × 5 cm each) extending from a central platform (5 × 5 cm). Each set of arms opposed one another and were enclosed by a 20-cm-high wall. The maze was elevated 40 cm above the ground. Each mouse was placed on the central platform with its head facing an open arm and was allowed to move freely for 5 min under dim light conditions (15 lux). The performance of the mouse for 5 min was videotaped using a digital camera, and then the following parameters were recorded and calculated: (i) time spent in various sections of the maze (open and closed arms, central platform); (ii) number of open and closed arm entries (arm entry defined as all four paws into an arm); (iii) ratio of open arm entries (open arm entries/total entries).

### Novel object recognition test

The object recognition test was performed according to a previous study.^28^ The task consisted of three sessions: habituation, training and retention. Each mouse was individually habituated to the cage (30 × 30 × 30 cm) with 10 min of exploration in the absence of objects for 3 days. Two wooden block objects were placed in the middle of the cage during each training session, and the mouse was allowed to explore the cage for 10 min. In the retention session, the mouse was placed back into the same cage with a novel wooden ball object together with a familiar wooden block object 6 h after the training session. The animal was then allowed to explore freely for 10 min and the time spent exploring each object was recorded. Exploratory behavior was defined as directing the nose to the object at a distance of <2 cm and/or touching it with the nose. Preference index, a ratio of the amount of time spent exploring a novel object over the total time spent exploring both objects in the retention session, was used to measure cognitive function.

### Social interaction test

The social interaction test was performed as previously described.^29^ Each mouse was placed in the observation cage and allowed 15 min of habituation under dim light (15 lux). After habituation, a juvenile (3 or 4-week-old) male C57BL/6 mouse (Shimizu Laboratory Supplies, Kyoto, Japan) was placed in the center of the cage, and the behavior of the mouse was recorded for 5 min. Total duration of social interactions (for example, sniffing, licking) was measured from recordings by a trained and blinded observer.

### Forced swim test

The forced swim test was performed as previously described.^25^ The mice were immersed in a glass cylinder containing 25 °C water for 6 min. Immobility was defined as no additional movements by the mouse beyond those that were required to keep the head above the water. The duration of immobility was measured from video recordings. After the test, the mice were dried thoroughly with a towel and returned to their home cage.

### PPI test

Acoustic startle responses in the pre-pulse inhibition (PPI) experiments were measured in a startle chamber (SR-LAB; San Diego Instruments, San Diego, CA, USA) using standard methods described previously.^30^ The testing session started with 5 min of habituation to the startle chamber in the presence of 65 dB background white noise. Testing was composed of a single pulse of 120 dB preceded five prepulses (40 ms) of 69, 73, 77 or 85 dB. Pulses were randomly presented with an average of 16 s between pulses. Ten no-stimulus trials were included to assess spontaneous activity during testing. PPI was calculated as a percentage score: PPI (%)=(1−[(startle response for pulse with pre-pulse)/(startle response for pulse alone)]) × 100.

### Statistical analysis

Adequate sample size was determined according to the previous studies that performed analogous experiments. The variances between the groups that are being statistically compared were similar. For animal studies, no randomization and blinding were used. Data are expressed throughout the manuscript as mean±s.e.m. Statistical analysis was performed using Statcel3 (OMS, Saitama, Japan) and JMP 10 pro (SAS Institute of Japan, Tokyo, Japan). Unpaired *t*-tests were applied to compare: expression levels of JMJD2B, JMJD2C, PSD95 (Supplementary Figure 3), Homer2, Rhob and Rhog (Supplementary Figure 6) in the hippocampus; spine number (Supplementary Figure 4b), structure (Supplementary Figure 4a), dendrite number and length in the hippocampus and somatosensory cortex. A two-sided test was applied, except for the analysis of spine number (one-sided test). The raw data applying *t*-test was normally distributed. The variance was similar between the groups that are being statistically compared. Two-way analysis of variance analysis and *post hoc* tests were applied to compare: Sholl analysis of dendrites of cultured hippocampal neuron total distance moved activity and rearing number. Mann–Whitney tests were applied to compare: decrease ratio of moved distance between the first 10 min and last 10 min of the open-field test, social interaction test, novel object cognition test, Y-maze test, elevated-plus-maze test (Supplementary Figure 5a), PPI (Supplementary Figure 5b), forced swim test (Supplementary Figure 5c), rhythm test (Supplementary Figure 5d) and SHIRPA test (Supplementary Table 2).

## Results

### JMJD2B expression in the central nervous system

**Figure 1:**
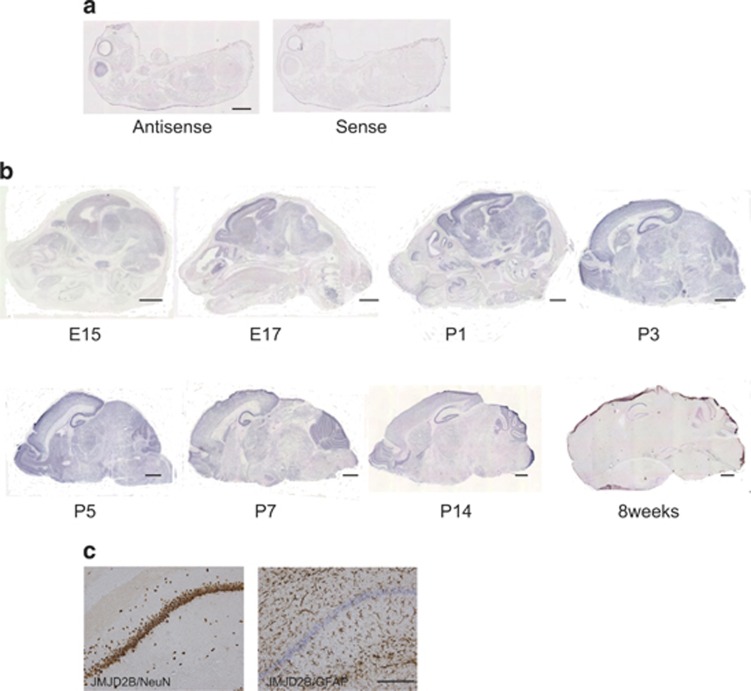
Expression of *Jmjd2b* in mouse. (**a**) *In situ* hybridization revealing expression of *Jmjd2b* mRNA in E17 mouse (left: antisense, right: sense; *n*=1). (**b**) *In situ* hybridization for the detection of *Jmjd2b* mRNA in the brain at the indicated stages (*n*=1 per age). (**c**) *In situ* hybridization for *Jmjd2b* mRNA, followed by immunostaining with NeuN (left) and GFAP (right; *n*=1). Scale bars: (**a**) 2 mm, (**b**) 1 mm, (**c**) 200 μm.

We first carried out *in situ* hybridization to characterize the expression of *Jmjd2b* mRNA in P0 WT mouse brain. Strong expression was observed in the brain and retina along with weaker expression in other organs (Figure 1a). We then analyzed expression of *Jmjd2b* mRNA in the brain at a series of developmental stages. From E15 to P14, *Jmjd2b* was rather diffusely expressed throughout the brain. Expression was especially strong in the cerebral cortex, hippocampus, basal ganglia, olfactory bulb and cerebellum. In 8-week-old mice, the expression level declined, but prominent signal could be observed in hippocampus and cerebellum. The time course of the expression of *Jmjd2b* mRNA throughout the brain is detailed in Figure 1b. In the adult hippocampus, JMJD2B mRNA was expressed in the CA1, CA3 and dentate gyrus. To assess the cell types expressing JMJD2B in the hippocampus, we performed immunostaining with DAB as the substrate for horseradish peroxidase using NeuN, GFAP and GAD67 primary antibody followed by *in situ* hybridization. The signal *for JMJD2B* mRNA was observed exclusively in NeuN-positive cells, but not in NeuN-negative cells (Figure 1c, left), and could not be detected in GFAP-positive astrocytes. These results demonstrate that *Jmjd2b* mRNA is predominantly expressed in neurons in the adult mouse hippocampus. Moreover the signal for *JMJD2B* mRNA was observed also in GAD67-positive cells (Supplementary Figure 1a). The signal for *JMJD2B* mRNA was observed in ganglionic eminence, where the interneurons are originated, in the brain on E13 (Supplementary Figure 1b). These results suggest that Jmjd2b mRNA is also expressed in GABAergic neuron.

### Generation of mice lacking JMJD2B in neurons

**Figure 2:**
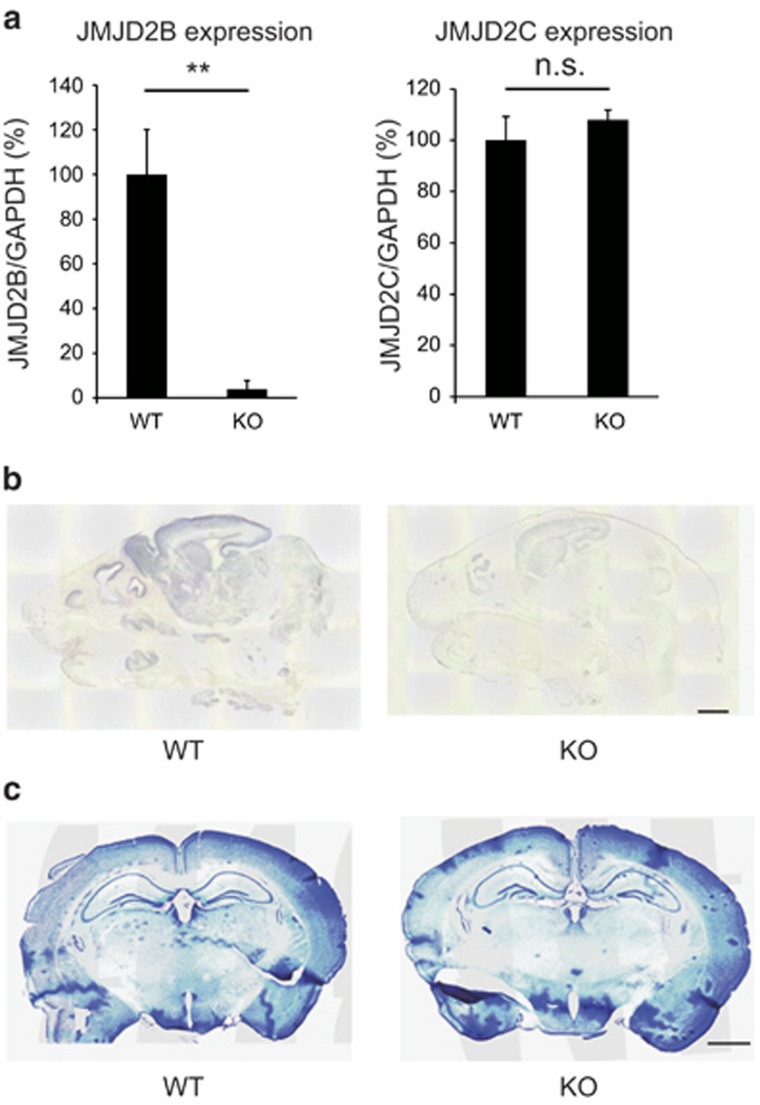
Generation of neuron-specific *Jmjd2b* mutant mice. (**a**) Relative expression level of *Jmjd2b* and *Jmjd2c*. The expression level was normalized to *Gapdh.* **P<0.01; NS, not significant. (WT, KO: *n*=3) Error bars represent s.e.m. (**b**) Expression of *Jmjd2b* mRNA in WT and KO mice. P0 mice were used for *in situ* hybridization. (WT, KO: *n*=1). (**c**) Nissl staining showing the gross morphology of the brain in the two genotypes. (WT, KO: *n*=3). KO, JMJD2B mutant mice; WT, wild-type mice. Scale bars: (**b**) 1 mm, (**c**) 50 μm.

We next explored the function of JMJD2B in neurons *in vivo* by generating mice lacking JMJD2B specifically in neurons. We crossed mice in which exon 5 of *Jmjd2b* was flanked by LoxP sequences biallelically (*Jmj2b flox/flox)*^13^ with mice expressing Cre recombinase under the tau promoter (*tau-Cre* mice),^21^ resulting in the deletion of coding regions necessary for demethylase activity of *Jmjd2b* specifically in the neurons of these compound mutant mice (*tau-Cre; Jmj2b flox/flox)*. JMJD2B gene expression was barely detectable in the brains of *tau-Cre; Jmj2b flox/flox* mice as determined by reverse transcription–PCR (*P*=0.009; Figure 2a, left). However, the expression level of *Jmjd2c*, a histone demethylase enzyme closely related to JMJD2B, was not changed in these mice (Figure 2a right). *In situ* hybridization for *Jmjd2b* confirmed deletion of this gene in these mice (Figure 2b). No obvious morphological differences were observed between the two genotypes including in the hippocampus (Figure 2c). For the remainder of the paper, we refer to mice lacking JMJD2B in neurons as JMJD2B mutant mice and exon 5-biallelic flanked mice as WT mice.

### JMJD2B regulates spine maturation in the hippocampus

Next, we investigated structures of the hippocampus in which JMJD2B was strongly expressed from embryonic stages through adulthood in detail. A previous report revealed that HDAC2, a histone modifier gene, affected spine number in hippocampus and regulated the formation of neural circuits.^31^ These findings prompted us to hypothesize that JMJD2B was associated with synaptic development. To assess this possibility, we examined the spine structure of hippocampal CA1 pyramidal neurons using the Golgi staining method.

**Figure 3:**
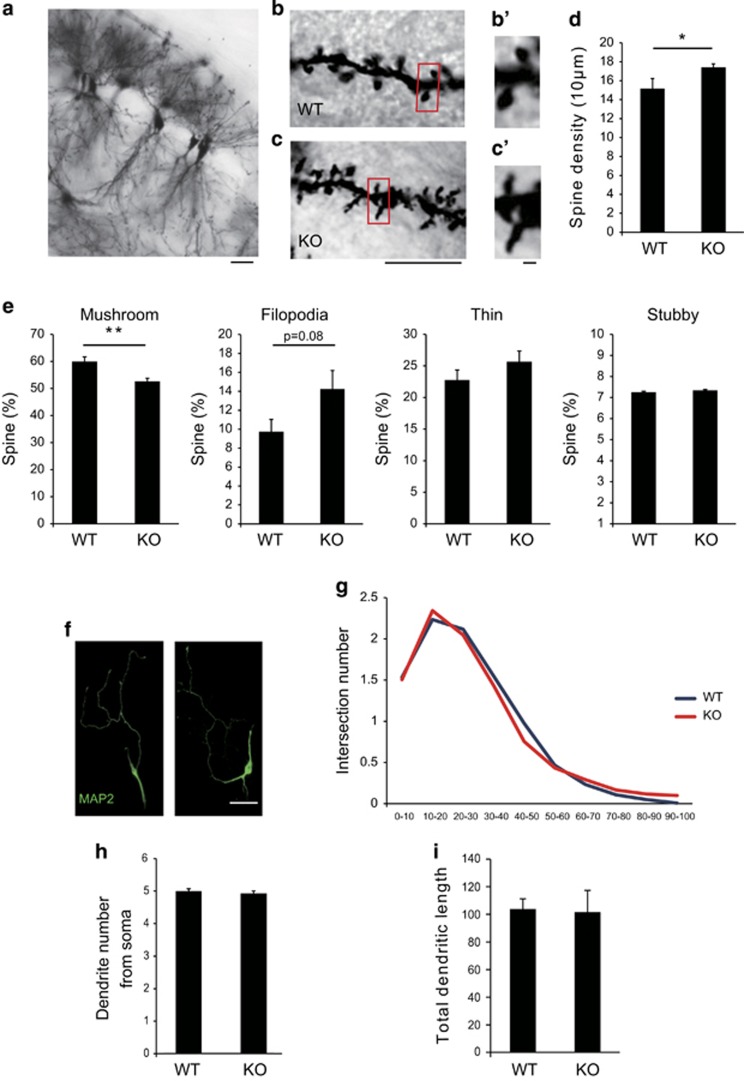
Structural analysis of dendrites and spines of hippocampal CA1 of JMJD2B mutant mice.(**a**) Representative Golgi staining image in the hippocampal CA1 region. (**b** and **c**) Representative Golgi staining images of dendritic spines in the hippocampal CA1 region in each genotype. (b′ and c′) Magnified images of **b** and **c.** (**d**) The total spine numbers in the CA1 region of the two genotypes. (**e**) The percentage of mature mushroom, filopodia, thin and stubby-type spines of CA1 pyramidal neurons. *P<0.05; **P<0.01. WT: *n*=6, KO: *n*=5 (**a–e**). Error bars represent s.e.m. (**f**; left) Representative images of hippocampal neurons cultured for 7 days, and stained with MAP2. (**g**) Sholl analysis of hippocampal neurons of each genotype. A concentric circle was drawn every 10 μm from a central focus on the neuronal soma and the number of intersections were counted. NS, not significant. (WT, KO: *n*=4). (**h**) The number of dendrites extending from the soma. (WT, KO: *n*=3). (**i**) Total dendritic length measured from the soma of cultured hippocampal neurons. Total dendritic length measured from the soma of cultured hippocampal neurons. (WT, KO: *n*=4). WT, wild-type mice; KO, JMJD2B mutant mice. Scale bars: (**a**) 10 μm, (**b** and **c**) 10 μm, (b'and c') 1 μm, (**f**) 20 μm. Error bars represent s.e.m. (**d**, **e**, **h**, **i**).

Dendritic spines of pyramidal neurons in the CA1 region receive direct and indirect excitatory inputs from various anatomical regions. Schaffer collaterals from CA3 are one of the major inputs to CA1 and they terminate in the distal two-thirds of the stratum radiatum of the CA1 region.^32^ Therefore, we analyzed the spine morphology and density on secondary dendrites of the pyramidal neurons in this area. We first counted the total number of spines per 20 μm dendrite and found a significant increase in the spine numbers in JMJD2B mutant mice (*P*=0.041; Figures 3a–d). We then performed structural analysis of spines, classifying them into mature spines (mushroom type) and immature spines (thin type, filopodia type and stubby type). We found that the number of mature mushroom type spines was significantly reduced in JMJD2B mutant mice (*P*=0.006), and that the number of immature filopodia type was increased (filopodia type; *P*=0.08; Figures 3a–c and e). However the expression level of PSD95 in hippocampus was not significantly different between the genotypes (Supplementary Figure 2).

As JMJD2B is expressed also in somatosensory cortex from late prenatal to early postnatal mouse, we analyzed the number and structure of spines in apical dendrite of layer 2/3 pyramidal neuron in somatosensory cortex. Notably, MeCP2 mutant mice, which display neurodevelopmental delay and spontaneous epileptiform activity, reveal increased number of spines in somatosensory cortex.^33^ However, we found no difference between the genotypes in either spine number or structure in somatosensory cortex (Supplementary Figure 3).

We next attempted to investigate the formation of dendrites *in vivo*. However, it was difficult to assess the length and elaboration of specific neurons, as the dendrites of Golgi-stained neurons overlapped with each other in the CA1 region. Therefore, we cultured hippocampal neurons derived from P1 JMJD2B mutant mice and WT mice for 7 days, and assessed the formation of dendrites *in vitro*. We found no significant differences in dendrite number, length or elaboration between the two genotypes (Figures 3f and i). We further examined whether abnormal mossy fiber sprouting was present in mutant mice using Timm staining and found no differences between genotypes (Supplementary Figure 4). Our findings therefore indicate that JMJD2B is required for normal spine formation in the hippocampal CA1 region, and disruption of JMJD2B gene leads to impaired dendritic spine maturation.

### JMJD2B mutant mice show hyperactive behavior

**Figure 4:**
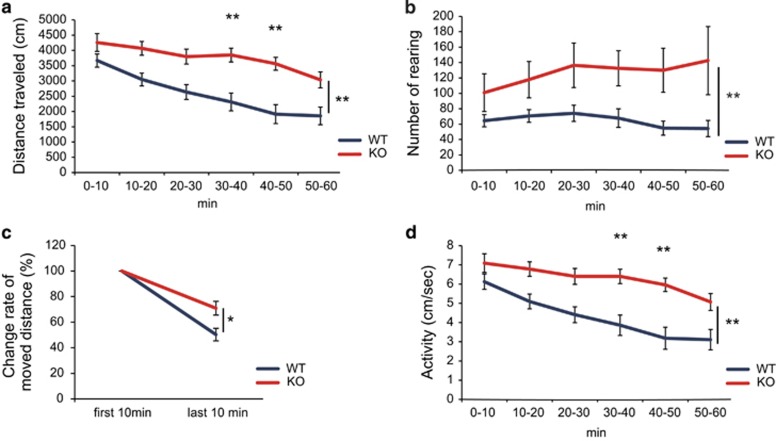
JMJD2B mutant mice reveal hyperactivity in the open-field test.(**a**) The graph shows the distance moved during the indicated time period. (**b**) The graph shows the temporal change in the rate of activity (speed). (**c**) The graph shows the distance moved in the first 10 min versus the last 10 min (50 to 60 min) of assessment. (**d**) The graph shows temporal changes in rearing number. (WT: *n*=12, KO: *n*=10). WT, wild-type mice; KO, JMJD2B mutant mice. *P<0.05; **P<0.01. Error bars represent s.e.m.

Defective histone demethylase function is associated with neurodevelopmental disorders. As our observations of impaired dendritic spine formation in JMJD2B mutant mice suggested that JMJD2B has an important role in the formation of neural networks in the CNS, we hypothesized that JMJD2B mutant mice might display abnormal behavior reminiscent of neurodevelopmental disorders. We therefore subjected JMJD2B mutant mice and their WT littermates to a comprehensive battery of behavioral tests (Supplementary Table 1). Mutant mice showed no obvious differences in their physical characteristics. On the other hand, JMJD2B mutant mice displayed significantly increased vocalization, which was the index of the excitability of mice^34^ (SHIRPA test; Supplementary Table 2). Spontaneous locomotor activity was assessed by the open-field test. There were significant differences between genotypes in the total distance traveled (between genotypes; *P*<0.0001, 30–40 min; *P*=0.0045, 40–50 min; *P*=0.0015; Figure 4a), rearing number (between genotypes; *P*=0.0027; Figure 4b) and activity (between genotypes; *P*<0.0001, 30–40 min; *P*=0.0048, 40–50 min; *P*=0.0012; Figure 4d).

We then tested their response to a novel environment, a test of anxiety and exploratory behavior. When WT mice were placed in a novel environment, they initially became hyperactive in response to the unfamiliar surroundings. They then became accustomed to the new environment and their enhanced activity gradually declined. However, JMJD2B mutant mice remained hyperactive even during the last 10 min of the test. Figure 4c shows the ratio of the distance moved in the last 10 min to the distance moved in the first 10 min (*P*=0.024).

Mice were also subjected to the elevated-plus-maze test, PPI test, forced swim test and sleep–wake rhythm test (Supplementary Figures 5a–d). There were no significant differences between genotypes except for a decrease of immobile time in mutant mice in the forced swim test (*P*=0.026). Although the forced swim test is used as an index of depressive-like behavior in mice, this result does not necessarily mean that the mutant mice show an anti-depressive tendency, as these mice show hyperactivity at baseline.

### JMJD2B mutant mice show deficits in working memory

**Figure 5:**
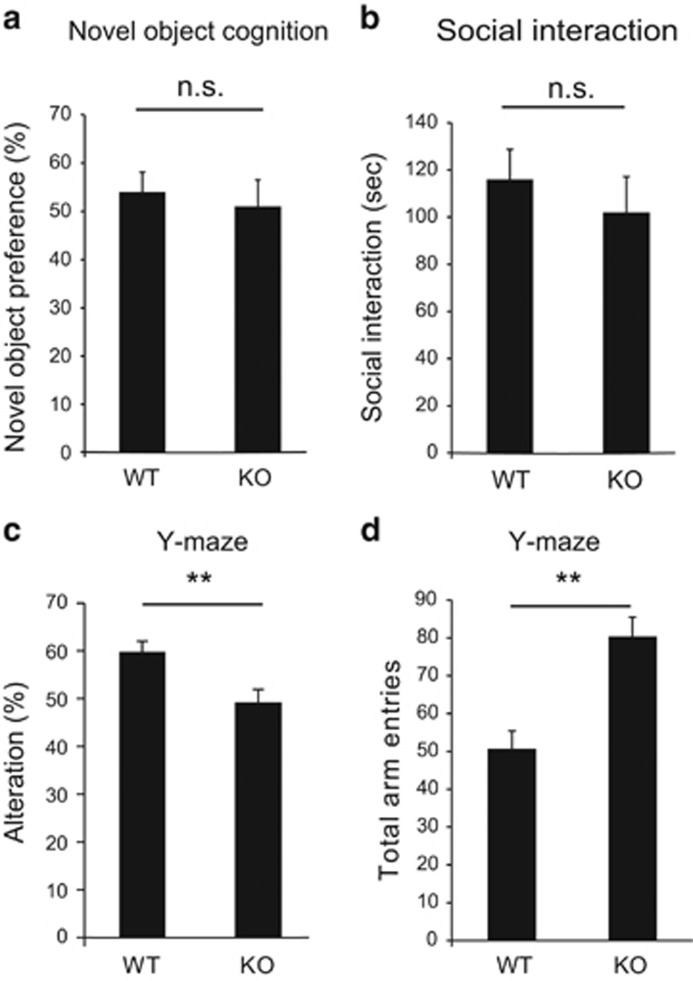
JMJD2B mutant mice show deficits in short-term memory. (**a**) The result of the social interaction test examining the social affinity to another mouse. The graph shows the relative time of exploration of the novel object. (WT: *n*=13 KO: *n*=11). (**b**) Novel object recognition test to examine the interest in unknown object. (WT: *n*=13 KO: *n*=11). (**c** and **d**) Y-maze test results examining short-term memory in the two genotypes. (WT: *n*=10; KO: *n*=11). (**c**) The graph shows the percentage of correct entries. (**d**) The graph shows the number of total entries. KO, JMJD2B mutant mice; NS, not significant; WT, wild-type mice. Error bars represent s.e.m. **P<0.01.

As two mutations of *JMJD2B* are found in individuals of ASD, we performed a series of experiments to assess autistic-like traits in these mice. First, an interest in novelties was tested in the novel object recognition test using a novel wooden ball object. There were no differences between genotypes in the exploration rate to a novel object (Figure 5a). Next, sociability was tested in social interaction test with a juvenile WT male mouse as a social partner. There were no differences between genotypes in the interaction time spent with a social partner (Figure 5b).

Previous reports have revealed that neurodevelopmental disorders are sometimes correlated with intellectual disability.^2^ Therefore, we examined whether working memory was impaired in JMJD2B mutant mice as assessed by spontaneous alteration behavior in the Y-maze task. Mice of each genotype were put into the box with a Y shape and the percentage of correct entry numbers per total entry numbers (alteration rates) was evaluated. WT mice exhibited alteration rates of approximately 60%. In contrast, JMJD2B mutant mice showed significantly reduced alteration rates compared with WT littermates (*P*=0.0074; Figure 5c). The total number of entries differed between genotypes, reflecting the hyperactivity of the mutant mice observed at baseline (*P*=0.0015; Figure 5d). These results suggest that working memory is impaired in JMJD2B mutant mice.

These results are in keeping with the aberrant neuronal morphology observed in JMJD2B mutant mice, as accumulating evidences indicate an association of memory function with alterations in dendritic spine morphology.^35^ Therefore, impaired hippocampal spine maturation may cause working memory deficits in JMJD2B mutant mice. Moreover, some JMJD2B mutant mice showed spontaneous epileptiform-like activity (data not shown), which is another important symptom of neurodevelopmental disorders.^1^ It occurred following stressful events such as cage opening, handling or placement in a novel environment. Seizures generally began at about 5 s after these events beginning with behavioral arrest and rigid motionless posture, followed by head and neck jerks, and clonus of forelimb and hindlimb. We observed such epileptiform-like activity in 3 mice out of 153 mutant mice in our experiment (~2%), though WT mice never displayed such activity. These mice that suffered from such epileptiform-like activity were all older than 8 weeks.

In summary, the JMJD2B mutant mice characterized in this study display several symptoms reminiscent of neurodevelopmental disorders.

## Discussion

In the present study, we analyzed the phenotype of JMJD2B mutant mice in brain development. We found a decreased number of mature spines and increased number of immature spines, resulting in increased total number of spines in JMJD2B mutant mice. Pyramidal neurons in the CA3 region partly transmit electrical signals to distal radial stratum of CA1 region through Schaffer collaterals. Therefore, it is intriguing to hypothesize that the abnormalities in spine number and structure in the CA1 region induce defective signal transmission from CA3 to CA1. The expression level of PSD95 in hippocampus was not changed. This is presumably because the change in the PSD95 expression in the hippocampal spines was region-specific or the minute change could not be detected by using the western blot test. JMJD2B mutant mice displayed spontaneous epileptiform-like activity. Although it is possible that seizure recurrence can alter synapse connectivity leading to change in spine number and structure in JMJD2B mutant mice, this would not be the case considering the rare incidence rate and age of onset of seizures (we used 8-week-old mice for analysis). A previous report suggests that JMJD2B is a causative risk gene for neurodevelopmental disorders,^8^ while accumulating evidence indicates that hippocampal spines in various mouse models of neurodevelopmental disorder are structurally and functionally abnormal.^36, 37, 38, 39^ For example, mice with a mutation in *SHANK2*, a risk gene for ASD, display an increased number of hippocampal spines and abnormal synaptic transmission in the hippocampus.^37^ Furthermore, high-density and spindle-shaped dendritic spines are also observed in the brains of individuals with neurodevelopmental disorders.^40^

We also observed behavioral alterations in JMJD2B-deficient mice. Recently, mutations of a number of genes involved in epigenetic modification, including DNA methylation, histone acetylation or deacetylation, and histone methylation and demethylation, have been identified in individuals with neurodevelopmental disorders. Indeed, two mutations of *JMJD2B* were identified in ASD patients. The results of open-field test suggest that JMJD2B-deficient mice are hyperactive at baseline and have trouble in becoming accustomed to a novel environment. Neurodevelopmental disorders sometimes correlate with intellectual disability.^2^ JMJD2B-deficient mice did in fact display working memory deficits as determined by assessment of correct entry number in the Y-maze test (Figure 5c). Moreover, these mice sometimes displayed epileptiform-like seizures, which are frequently observed in neurodevelopmental disorders.^3^ These mice also displayed anti-depressive-like behavior in the forced swim test. However, we should be careful in the interpretation of this data, as this tendency may just reflect the heightened baseline hyperactivity of these mice. On the other hand, these mice did not display excessive anxiety-like or ASD-like behaviors, such as social interaction deficits or indifference to novel objects, or other abnormalities (PPI and rhythm tests).

Taken together these behavioral data indicate that JMJD2B mutant mice display attention deficit hyperactivity disorder-like behavior with memory deficits and epilepsy. However, *JMJD2B* is suspected to be a risk gene for ASD. One possible reason for this discrepancy is that our behavioral tests may not be appropriate for the detection of ASD-like symptoms in this mouse. For example, several mouse models with mutated risk genes for ASD display abnormalities in ultrasonic vocalization, and this important behavioral characteristic in ASD-model mice was not tested in our study.^41, 42^ Although rearing behavior in mice is generally described as an index of exploratory behavior, increases in the frequency of rearing number observed in JMJD2B mutant mice may replicate repetitive behavior in ASD. In fact, recent reports have revealed that Shank3 mutant mice, a model of ASD, display enhanced rearing.^43^

JMJD2B is a histone lysine demethylase that acts as a transcriptional activator through demethylation of H3K9me3. Therefore, the transcription of target genes is suppressed when JMJD2B is depleted. Hippocampal spine formation, elimination and maturation are dynamic processes that maintain the synaptic network of the brain. Actin-binding proteins, postsynaptic scaffolding proteins and low-molecular-GTP-binding proteins have been reported to be important factors involved in these processes.^44^ Transcriptional silencing of such factors by JMJD2B depletion may alter their expression level, which may in turn underlie the increase in total spine number with a decrease in mature spines that we observed in the hippocampal CA1 region of JMJD2B mutant mice. A previous report demonstrated that G9a histone methyltransferase elevates the density of dendritic spines in nucleus accumbens neurons by reducing H3K9me2 levels on exposure to cocaine.^45^ In addition, mutation of *MeCP2*, the causative gene of Rett syndrome and an epigenetic regulator that acts by binding to methylated DNA, also causes a decrease in the number of dendritic spines.^46^ Thus, there is increasing evidence that epigenetic mechanisms affect the development and plasticity of dendritic spines. A differential genome-wide expression study for JMJD2B-deficient ES cells reported that gene expression of several genes involved in synaptic function such as Homer2, Rhob and Rhog was changed.^47, 48, 49^ However, the expression level of these genes was not changed in hippocampus of JMJD2B mutant mice in our experimental paradigm (Supplementary Figure 6). Further studies are needed to identify the target genes of JMJD2B. However, it should be noted that the genes involved in the regulation of spine structure might not be direct targets of JMJD2B.

It has been shown that various regions in the brain are affected in the neurodevelopmental disorders. For example, pathological findings are observed in cerebral cortex, amygdala, basal ganglia, and cerebellum and hippocampus in neurodevelopmental disorders.^50, 51^ Most notably, abnormal neurogenesis in the cerebral cortex is associated with ASD-like behavior.^52^ Indeed, disorganization of the cerebral cortex is observed in children with ASD.^53^ JMJD2B, which is strongly expressed in the cerebral cortex at perinatal stages, might also play a role in cortical neurogenesis. It is possible that the deficiency of JMJD2B causes abnormal cerebral neurogenesis resulting in abnormal behavior. Further research is needed to examine whether the hippocampus is the region responsible for abnormal behavior in JMJD2B mutant mice.

In conclusion, our study elucidates the function of JMJD2B in the dendritic spine regulation and behavior, and provides a new mouse model for neurodevelopmental disorders.
