# Supplementary material for: Deletion of JMJD2B in neurons leads to defective spine maturation, hyperactive behavior and memory deficits in mouse
Source: Transl Psychiatry. 2016 Mar 29;6(3):e766–. doi: 10.1038/tp.2016.31 (PMC4872455; doi:10.1038/tp.2016.31)

**Supplementary Information**

**Supplementary Figure 1. Expression of JMJD2B in interneuron**

(a)*In situ* hybridization for Jmjd2b mRNA, followed by immunostaining for GAD67 (n=1) (b) (Right) *In situ* hybridization for Jmjd2b mRNA in the brain at embryonic days 13 mouse (Left) Magnified image of medial ganglionic eminence. (n=1) Scale bars; (a) 20μm, (b) 200μm (Left) 80μm (Right)

**Supplementary Figure 2. The expression of PSD95 in hippocampus**

The expression of PSD95 was not changed between the genotypes（WT: n=3, KO: n=3）

**Supplementary Figure 3. Structure and number of spines in somatosensory cortex**

(a) The percentage of mushroom, filopodia, thin, and stubby spines of dendrites in somatosensory cortex (b) The number of spines in somatosensory cortex between the genotypes. (WT, KO: n=3) n.s., not significant. Error bars represent s.e.m.

**Supplementary Figure 4. Mossy fiber morphology observed by Timm staining**

The morphology of mossy fibers appears to be normal in JMJD2B mutant mice. WT, wild-type mice; KO, JMJD2B mutant mice. (WT: n=3, KO: n=3) Scale Bar: 100 μm.

**Supplementary Figure 5. The results of additional behavioral tests**

(a) The results of the elevated plus maze test to examine anxiety. The graph shows the ratio of the time in open arm to total time. (WT: n=13 KO: n=11) (b) The results of the pre-pulse inhibition test. The graph shows the pre-pulse inhibition rate at various pre-pulse volumes. (WT: n=10 KO: n=11) (c) The results of forced swim test to assess depression-like behavior.. The graph shows immobility time per trial. (WT: n=12 KO: n=11) (d) (Left) Time schedule of

the rhythm test. (Right) The graph shows the ratio of activity in the dark phase to total activity. (WT: n=8 KO: n=8) *, $P < 0.05$; **, n.s., not significant. Error bars represent s.e.m.

**Supplementary Figure 6. The expression of Homer2, Rhob, and Rhog in hippocampus**

(a,b,c) The expression of Homer2, Rhob and Rhog in hippocampus was not changed between the genotypes. (WT: n=3 KO: n=3) n.s., not significant. Error bars represent s.e.m.

**Supplementary Table 1. Time schedule of behavioral test battery**

Each test was performed after an interval of at least 3 days from the previous test.

**Supplementary Table 2. The results of SHIRPA test**

The table shows the contents of SHIRPA test and the difference between genotypes. (WT: n=13 KO: n=11) (n.s., not significant)

Supplementary figure 1

a

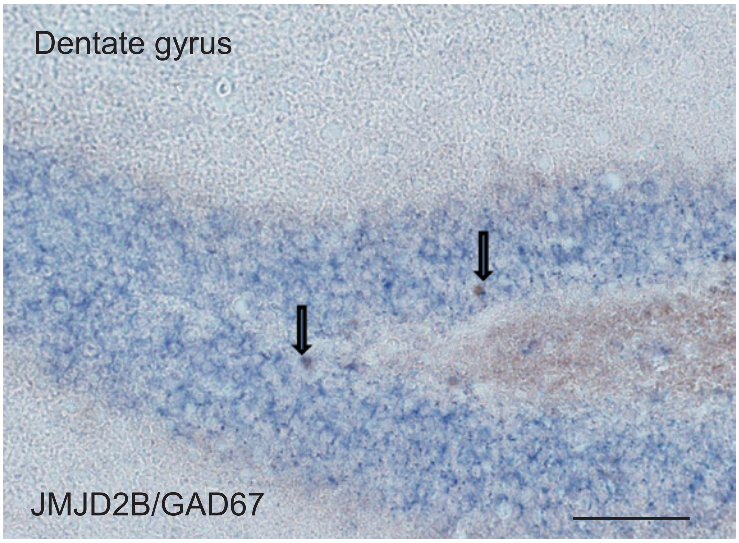


b

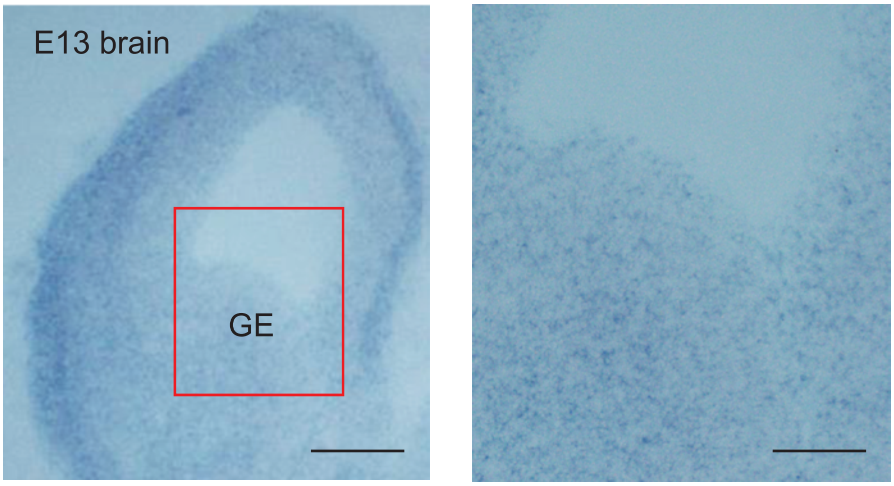

Supplementary Figure 2

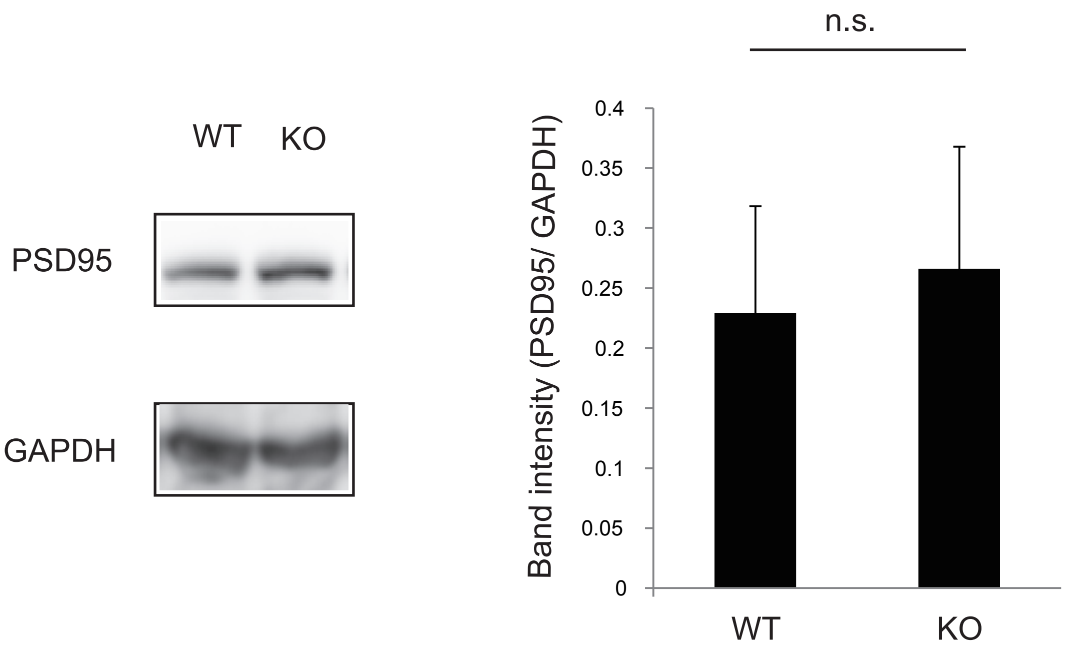

Supplementary Figure 3

Cortex

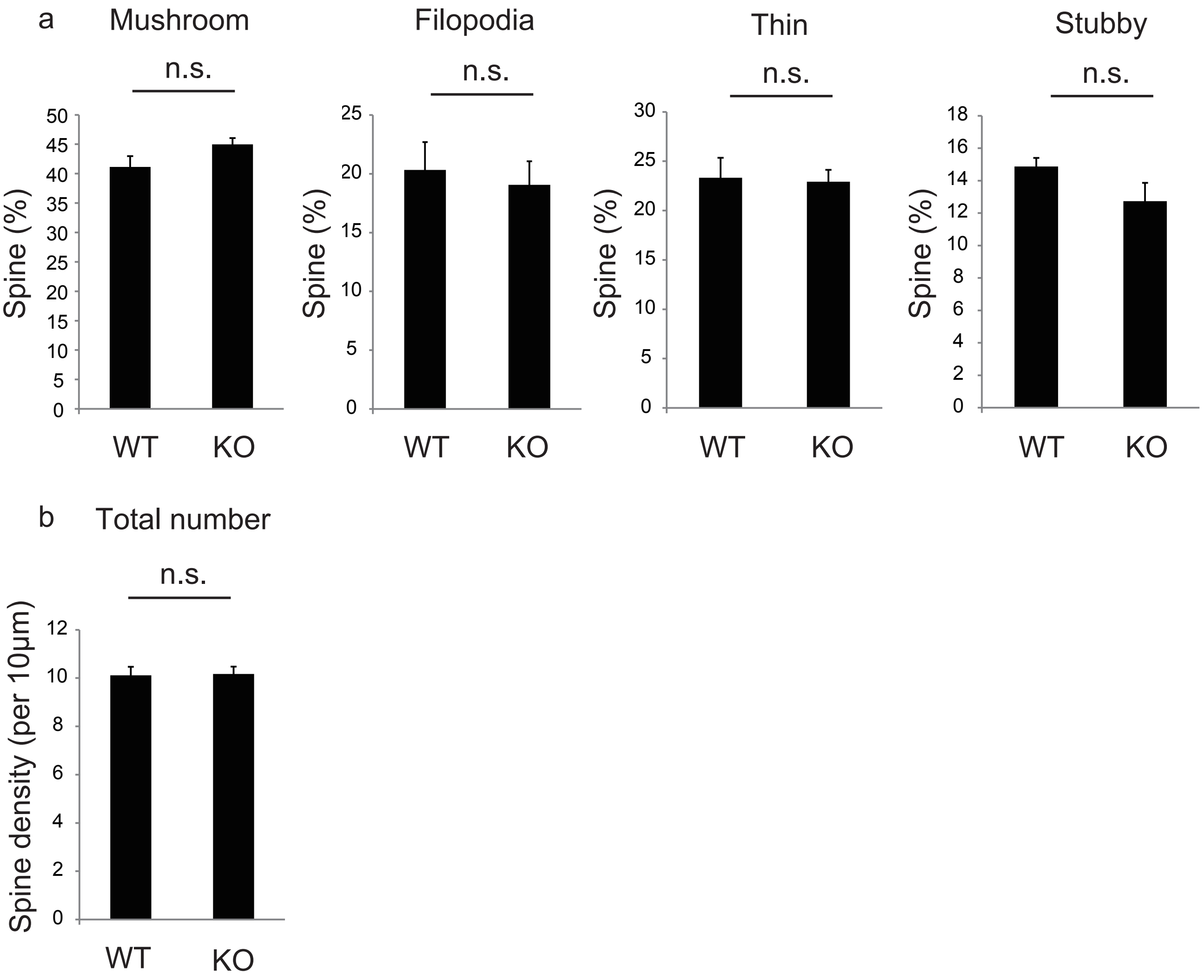

Supplementary Figure 4

WT

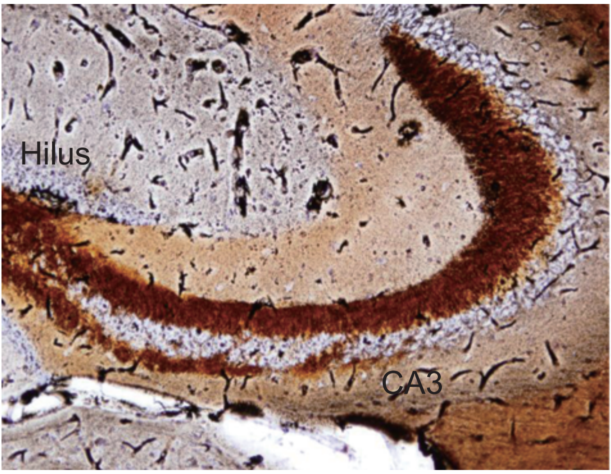


KO

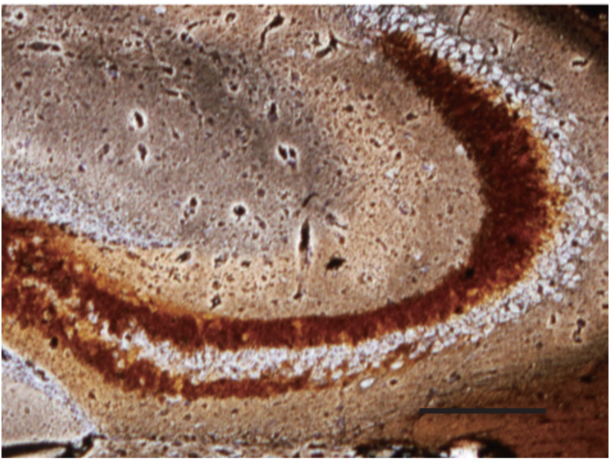

Supplementary Figure 5

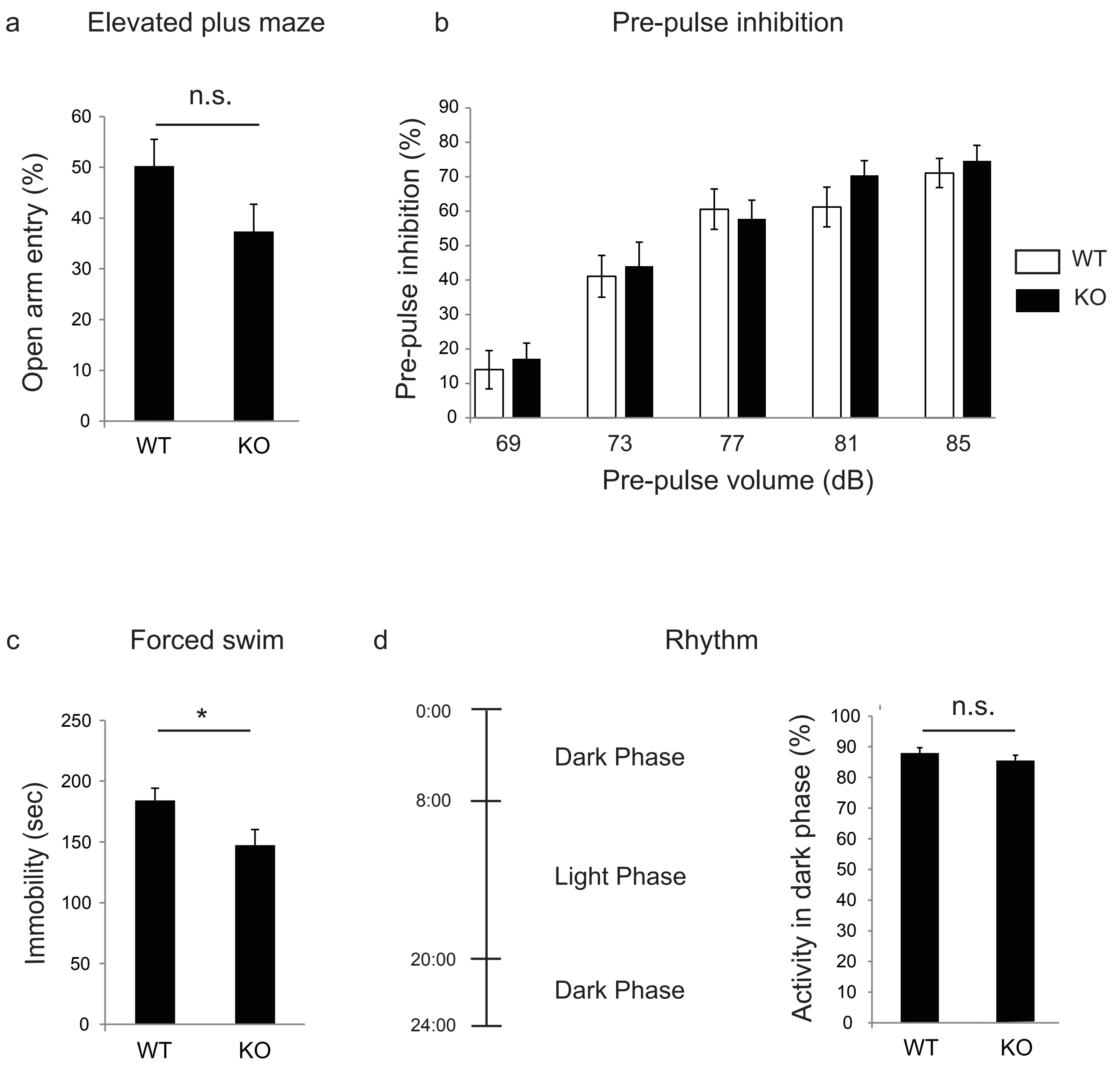

Supplementary Figure 6

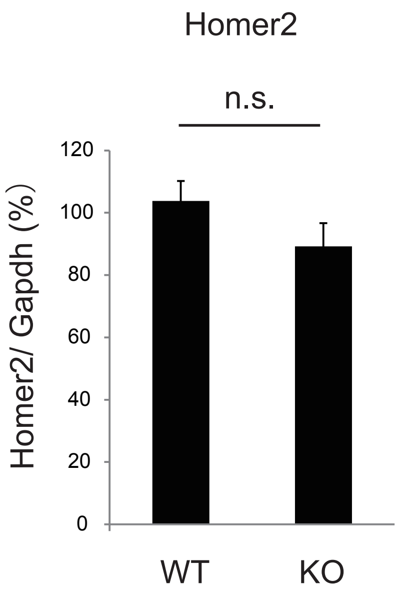


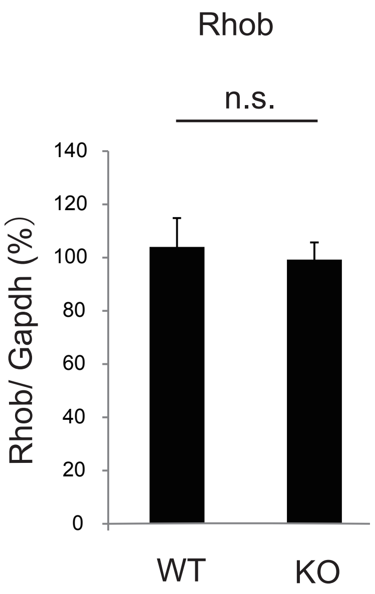


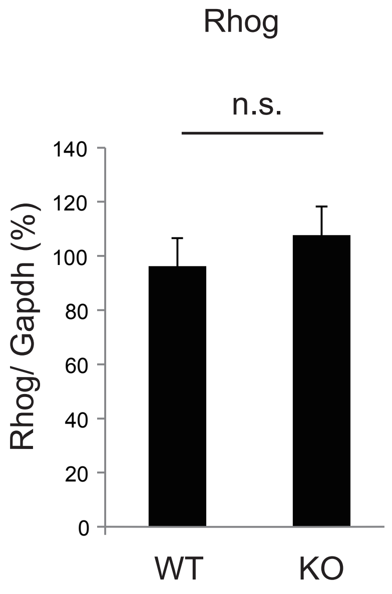

Supplement: Supplementary Figures [file tp201631x1.pdf]
