# Supplementary figures and images for: Deletion of JMJD2B in neurons leads to defective spine maturation, hyperactive behavior and memory deficits in mouse
Source: Transl Psychiatry. 2016 Mar 29;6(3):e766–. doi: 10.1038/tp.2016.31 (PMC4872455; doi:10.1038/tp.2016.31)

Supplementary table1

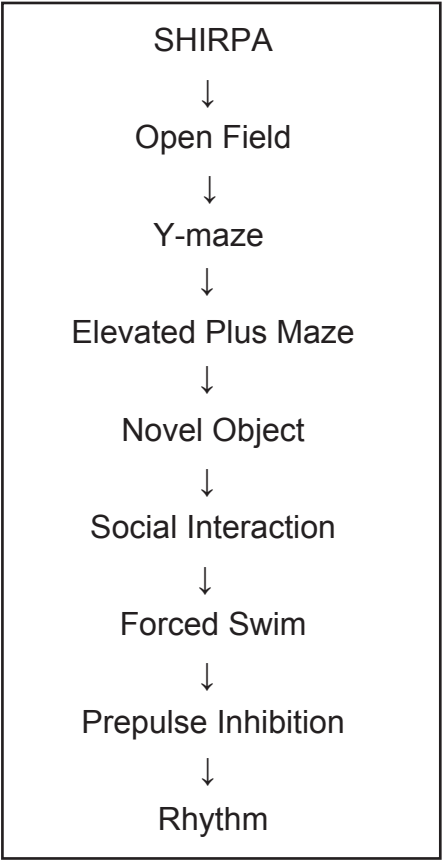

Supplement: Supplementary Table 1 [file tp201631x2.pdf]
