# Supplementary material for: Deletion of JMJD2B in neurons leads to defective spine maturation, hyperactive behavior and memory deficits in mouse
Source: Transl Psychiatry. 2016 Mar 29;6(3):e766–. doi: 10.1038/tp.2016.31 (PMC4872455; doi:10.1038/tp.2016.31)
Supplement: Supplementary Table 2 [file tp201631x3.pdf]

Supplementary Table 2

No.	Assessment	f/f	f/f taucre	significant difference
1	coat color	0.0	0.0	n.s.
2	hair length	0.2	0.2	n.s.
3	hair morphology	0.0	0.0	n.s.
4	respiration rate	2.0	2.0	n.s.
5	tremor	0.0	0.0	n.s.
6	body position	2.8	2.9	n.s.
7	spontaneous activity	2.4	2.6	n.s.
8	defecation	3.2	2.3	n.s.
9	urination	0.3	0.2	n.s.
10	latency to move	6.4	11.0	n.s.
11	locomotor activity	10.7	13.4	n.s.
12	transfer arousal	3.4	3.4	n.s.
13	piloerection	0.0	0.0	n.s.
14	palpebral closure	0.1	0.0	n.s.
15	startle response	0.9	1.0	n.s.
16	gait	0.0	0.0	n.s.
17	pelvic elevation	1.9	2.0	n.s.
18	tail elevation	1.0	1.0	n.s.
19	touch escape	1.7	2.0	n.s.
20	positional passivity	0.1	0.0	n.s.
21	trunk curl	0.0	0.0	n.s.
22	limb grasping	0.0	0.0	n.s.
23	visual placing	2.1	2.0	n.s.
24	grip strength	2.5	2.6	n.s.
25	body tone	0.3	0.5	n.s.
26	head morphology	0.0	0.0	n.s.
27	pinna reflex	0.9	1.0	n.s.
28	pinna morphology r	0.0	0.0	n.s.
29	pinna morphology l	0.1	0.1	n.s.
30	corneal reflex	1.0	1.0	n.s.
31	toe pinch	0.4	0.4	n.s.
32	body length	8.5	8.3	n.s.
33	tail length	6.9	6.9	n.s.
34	tail morphology	0.1	0.1	n.s.
35	lacrimation	0.0	0.0	n.s.
36	whisker morphology	0.1	0.1	n.s.
37	teeth morphology	0.0	0.0	n.s.
38	provoked biting	0.1	0.5	n.s.
39	salivation	0.0	0.0	n.s.
40	heart rate	1.1	1.5	n.s.
41	abnormal tone	0.5	0.5	n.s.
42	skin color	1.4	1.5	n.s.
43	limb morphology fr	0.0	0.0	n.s.
44	limb morphology fl	0.0	0.0	n.s.
45	limb morphology fr	0.0	0.0	n.s.
46	limb morphology fl	0.0	0.0	n.s.
47	limb tone	0.2	0.1	n.s.
48	wire manover	0.2	0.0	n.s.
49	righting reflex	0.0	0.0	n.s.
50	contact righting reflex	0.8	0.9	n.s.
51	negative geotaxis	0.1	0.1	n.s.
52	fear	0.0	0.0	n.s.
53	irritability	0.8	0.8	n.s.
54	aggretion	0.4	0.3	n.s.
55	vocalization	0.7	1.0	p=0.0405
56	body weight	25.4	25.0	n.s.
